# Cognitive Interpretation Bias: The Effect of a Single Session Moderate Exercise Protocol on Anxiety and Depression

**DOI:** 10.3389/fpsyg.2018.01363

**Published:** 2018-08-08

**Authors:** Séraphine C. Clarke, Nicholas R. Cooper, Mirinalee Rana, Bundy Mackintosh

**Affiliations:** Department of Psychology, Centre for Brain Sciences, University of Essex, Colchester, United Kingdom

**Keywords:** anxiety, depression, ameliorating affect, mood enhancement, cognitive interpretation bias, physical exercise

## Abstract

Research conducted within the cognitive bias modification (CBM) paradigm has revealed that cognitive biases such as negative cognitive interpretation biases contribute to mental health disorders such as anxiety (Beard, 2011). It has been shown that exercise reduces anxiety (Ensari et al., 2015). Exercise has also been found to reduce negative cognitive attention biases (Tian and Smith, 2011), however, no research to date has investigated the effect of exercise on cognitive interpretation bias. The key aims of the current project is to investigate whether moderate exercise reduces self-reported symptoms of depression and stress. Additionally, to establish which intensity of exercise is required to achieve anxiety reduction and reduce an individual’s negative cognitive interpretation biases. Study 1 recruited a healthy sample of adult participants who were randomly assigned to one of two conditions: a walking exercise protocol or a control condition (*n* = 2 × 12). Participants completed anxiety and cognitive interpretation bias measures before and after the walking exercise or control condition. Those in the walking exercise condition presented less symptoms of trait anxiety on a measure of state and trait anxiety inventory (STAI), compared to controls relative to baseline measures following the intervention. Study 2 recruited frequent exercisers who were assigned to an exercise or control group (*n* = 2 × 24). Participants completed anxiety, depression, psychological stress, and cognitive interpretation bias measures before and after the exercise or control condition. Following the intervention, negative interpretation biases decreased in the exercise group and stayed stable in the control group. The exercise group also had significantly decreased anxiety, depression, and stress measures after the exercise condition, while controls did not. The research concludes that CBM holds promise for the management of mood disorders and exercise is an effective accompaniment to psychotherapy.

## Introduction

The beneficial effects of acute and chronic exercise on healthy individuals and those with sub-clinical or clinical anxiety and depression have been well established (Herring et al., 2014; Stubbs et al., 2017) and exercise has been advocated as a treatment for maladaptive mood and emotional problems (Paluska and Schwenk, 2000; Barbour and Blumenthal, 2005; Penedo and Dahn, 2005; Otto et al., 2007). Health care providers such as the National Health Service (NHS) in the United Kingdom have advocated exercise interventions independent or in conjunction with psychological and/or pharmacological therapies for affective emotional problems (Fox, 1999; Saxena et al., 2009; Daley, 2018). Benefits of physical exercise in a variety of different physical disorders including diabetes, renal disease, and osteoporosis, has been shown to be effective (Fentem, 1994). There is also a strong body of evidence regarding the physiological benefits of regular physical exercise on the human body (Salmon, 2001; Asmundson et al., 2013), as well as improved psychological wellbeing (Fox, 1999). Exercise is arguably not a panacea for mental health problems, however, it can reduce the symptoms of the less severe cases of anxiety, chronic stress and depression, and in turn could reduce rising health costs nationally and internationally (McCrone et al., 2008).

Although anxiety disorders are characterized particularly by symptoms of worry, the basis of vulnerability and fundamental mechanisms are common in all anxiety disorders (Mineka et al., 1998). The study of the cognitive dimensions of anxiety focuses on this notion of vulnerability (Clark and Beck, 2011). Vulnerability is defined as an individual’s perception of the ‘self’ that is exposed to internal and external threat over which the individual has little or no control to provide a reassurance of security or safety (Beck et al., 2005). Also, in the previous 30 years or so, cognitive models of anxiety and depression disorders have greatly highlighted the fundamentally crucial part selective information processing (so-called ‘cognitive bias’) plays in the development and maintenance of emotional psychopathology (Beck and Clark, 1997). However, the effects of physical exercise on the processing of these threat-related negative cognitive biases in individuals who experience frequent and severe episodes of anxiety and anxiety disorders is much less understood (Cooper and Tomporowski, 2017).

Physical activity and physical exercise are definitions that are usually used interchangeably, however, these concepts are not the same. Physical activity is defined as any bodily movement producing the expending of energy. While physical exercise is a subset of physical activity that is routine and structured with the aim to improve cardiovascular fitness level and overall health and wellbeing (Caspersen et al., 1985). Research has primarily focused on structured physical exercise programs relative to its psychological benefits, although there has been an emergence of cross-sectional studies which have consistently shown high self-reported levels of habitual physical activity to be associated with better mental health (Salmon, 2001). Research into the effects of exercise on emotion is predominantly focused on self-reported measures of anxiety, stress and depression symptoms or on clinical psychological assessment measures, to investigate the effects of exercise after a single protocol of exercise, a training program or affective responses during physical exercise (Williams et al., 2008; Ekkekakis, 2013). These experiments have undoubtedly given insights on benefits of exercise on alleviating moods and there is substantial evidence for this relationship to be considered as a therapeutic approach in the treatment of anxiety disorders (Fox, 1999). Previous research by Hansen et al. (2001) and Salmon (2001) suggest that exercise is emotion enhancing when it is performed at a ‘manageable level’ for the individual that is engaged in the activity.

Clinical and subclinical levels of anxiety are associated with biases in the predominant and automatic strategic stages of attention (Bar-Haim et al., 2007), while depression is associated with cognitive biases in the later stages (Gotlib et al., 2004; Joormann and D’Avanzato, 2010; Hallion and Ruscio, 2011). However, depression and anxiety are highly comorbid (Brown et al., 2001) and anxiety has also been associated with negative cognitive interpretation bias as well as attentional biases (Mathews and MacLeod, 2005; Hallion and Ruscio, 2011). This proposes another understanding of anxiety, perhaps it is characterized by attentional bias, the tendency to search for high risk, threatening or danger to the self, and also interpretation biases which is a tendency to evaluate present and past situations in negative, rather than benign or positive, manner. While depression may only be characterized by interpretation biases, which would explain why individuals with depression commonly ruminate about situations from the past (Krahé et al., 2016). Furthermore, the psychotherapy interventions in place for anxiety and depression disorders are grounded in cognitive theory that rest heavily on the assumption that cognitive biases are causally related to symptoms. These assumptions have been demonstrated and a strong relationship between anxiety, depression and a variety of negative cognitive biases (attentional, interpretational, and memory) is becoming established in the literature (Mathews and MacLeod, 2005; Vrijsen et al., 2014). Individuals who suffer with anxiety or depression are therefore likely to have negative cognitive interpretation biases (Richards and French, 1992; Richards, 2004).

Cognitive models of the functioning of anxiety suggest that there are biased routes of our fundamental information processing, operating within our cognitive system that possibly are not consciously accessible and these are important in experiencing an unexpected manifestation of anxiety (Mathews and MacLeod, 2005). It is suggested that individuals who present with anxiety and depression tend toward the negative aspect of ambiguous situations and therefore inclined to interpret such ambiguous and neutral conditions negatively instead of positively. Negative mood states are frequently related to negative cognitive biases of attention and interpretation (Rose and Parfitt, 2007). Individuals who suffer with clinical disorders such as anxiety, stress and depression, frequently exhibit a preferential response toward negative relative to positive or benign/neutral information and have a tendency to interpret ambiguous situations and environments negatively rather than positively. There is evidence to suggest that cognitive biases are rooted in psychological processes in clinical populations; suggesting that anxiety and stress-related symptoms could be considerably reduced in vulnerable populations if we could reduce their tendency to make negative cognitive biases and thereby improve mood state (Mackintosh et al., 2006; Brosnan et al., 2011). In terms of cognitive bias, depression and anxiety are associated with difficulty in disengaging one’s attention from mood-congruent (negative) self-relevant stimuli. This may be coupled with attentional avoidance to positive stimuli. Together, these lead to a decrease in positive mood and increase in negative depressive symptoms (Gotlib et al., 2004; Mathews and MacLeod, 2005).

Research conducted by Salmon (2001), suggests attention to aerobic exercise has been unproportionate to the focus given to non-aerobic exercises in which muscle activity is brief, high intensity and is not maintained. Aerobic physical exercise can be defined as an activity involving large muscle groups, increased heart rate and respiration, such as swimming, running, and aerobic dancing. According to a meta-analysis by Petruzzello et al. (1991), there were no significant disparities between the types of aerobic exercise an individual engaged in, the only significant differences were aerobic physical exercise having an increased positive effect on mood than non-aerobic exercises. These studies consistently show an improvement in mood after engaging in an aerobic exercise protocol. However, a study by Gupta et al. (2006) investigated the effect of a non-aerobic short-term yoga program on individuals with various mental and physical health issues, specifically anxiety. Their findings suggest the yoga program significantly reduced participants self-reported state and trait anxiety levels from by the end of the program. This suggests that some non-aerobic exercise can have a positive effect on reducing self-report anxiety, in this case specifically for a clinical sample.

Smith (2013) investigated the role of moderate exercise on emotional affect, using an exercise protocol and the perceived rated level of exertion scale (PRE) to achieve a moderate amount of exercise enough to be deemed beneficial. Smith (2013) found that moderate exercise reduced levels of state anxiety and concluded that moderate exercise has anxiolytic effects and, furthermore, could be resistant to emotionally arousing negative/threatening stimuli. This has implications for how positive mood could be potentially protected by moderate exercise even when individuals are presented with negative imagery. This effect of emotional resilience has also been reported by O’Connor and Shimizu (2002).

Research into the effects of exercise training on cognitive interpretation biases in anxiety, depression, and stress-related disorders is sparse and the insight it could lend to our understanding of the cognitive mechanisms could be valuable. The key aims of the present study is to establish which intensity of exercise is required to achieve anxiety reduction and whether this is related to changes in an individual’s negative cognitive interpretation biases. Furthermore, it will investigate whether there will be a reduction in anxiety when exercise is performed and reduces self-reported symptoms of depression and stress. These research aims will be addressed with two experimental studies. The hypothesis of Study 1 is that there will be significant decrease in negative interpretation bias while under a cognitive load (remembering a six-digit number during the task) compared to when not under a cognitive load and a reduction in anxiety measures in the walking exercise condition relative to the control condition.

## Study 1

### Methodology

#### Participants

Twenty-four participants were recruited to take part in this study from a student population at the University of Essex, Colchester, United Kingdom (see **Table 1** for mean age, gender ratio, and mean HR rest). To be eligible, individuals had to be able to engage in physical activity. Study requirements were that individuals did not exercise for 12 h prior to participating and must be fluent in spoken and written English. Participants were told they would be required to complete a series of questionnaires, and they may or may not be required to undertake a walking exercise protocol. Ethical approval was granted from the University of Essex Ethics Committee.

**Table 1:** Study 1: Mean and standard deviation for walking and control condition for STAI and SST measures.

	Baseline: Session 1 Post condition: Session 2	*N*	Mean, range, and *SD* age	Gender ratio M:F	Walking exercise condition Mean (*SD*)	Control condition Mean (*SD*)
STAI-S	Session 1	12	Walking Exercise Group	Walking Exercise Group: 1:5	37.58 (9.22)	33.25 (9.88)
			Mean: 29.50			
			Range: 21–40			
			*SD*: 7.03			
	Session 2				30.50 (8.74)	31.08 (941)
STAI-T	Session 1	12			44.0 (13.78)	38.5 (9.51)
	Session 2				40.41 (15.69)	38.16 (11.18)
SST Cognitively loaded	Session 1	12	Control GROUP:	Control GROUP: 2:4	30.87 (27.67)	19.64 (19.74)
	Session 2				31.18 (28.08)	14.96 (13.81)
			Mean: 28.58			
			Range: 22–38			
			*SD*: 5.45			
SST Cognitively NON-loaded	Session 1	12			26.58 (23.19)	19.50 (15.37)
	Session 2				31.16 (29.08)	22.56 (21.91)


#### Materials

##### State and trait anxiety

The state and trait components of the State Trait Anxiety Inventory (STAI; Spielberger, 1983) questionnaire were used to assess global and transient levels of anxiety. The STAI assesses apprehension, tensions, nervousness, and worry in 20 questions on a 4-point Likert scale. The first part measures state anxiety; which is an immediate measure of the participant’s current state of anxiety. The second part is the trait anxiety scale, which measures an individual’s predisposition for personal anxiety. This scale has been identified as a reliable tool for assessing anxiety and an individual’s aggregate. Reliability scores of this scale range from 0.65 to 0.86 for the trait version and 0.16 to 0.62 for the state version (Barnes et al., 2010).

##### Cognitive interpretation bias

The Scrambled Sentences Test (SST; Rude et al., 2002; Standage et al., 2010) was used to measure negative cognitive interpretation bias. The SST involves unscrambling a list of 20 sentences, this is done in cognitively non-loaded and cognitively loaded conditions, where the participant is subject to a simultaneous cognitive load task or not (e.g., remembering a six-digit number). Participants firstly, had the task explained to them by the researcher and then were asked to complete three practice questions, which were scored before the participant completed the SST to ensure they understood the task correctly, the practice question scores were not recorded for further analysis. For the loaded and non-loaded trials, only those during which the participant correctly recalled the six-digit number were included for analysis. Participants had 4 min to complete as many of scrambled sentences as possible. The score of negative or positive responses to the cognitive interpretation bias tasks were then collected and recorded. This method aims to investigate the tendency of each individual to interpret ambiguous information either positively or negatively and was measured before and after the intervention. From the data gathered, a negative interpretation bias score was calculated using Eq. (1). The SST has been suggested to be reliable at establishing a cause and effect between negative interpretation biases and predicting there after depressive symptoms for up to 8 weeks post SST (Rude et al., 2002).

(1) $$SST=100\times \frac{Negative}{Correct}$$

#### Design

Anxiety measures are assessed using a 2 × 2 mixed factorial design in which the within-subjects factor is Time (Session 1 and Session 2) and the between-subjects factor is Intervention group. The SST data is analyzed with a mixed measures 2 × 2 × 2 factorial design: SST-type (Load/NoLoad) × Session (Session 1 and Session 2) × Intervention (walking exercise/Control Group). The within-subjects variables are SST-type, and Session, while the between-subjects factor is the intervention (walking exercise) or control group.

#### Procedure

Once participants were recruited and deemed eligible for the study, they were invited to a study appointment with the primary researcher. The researcher summarized the process of the experiment and what would be required of them for participation. Participants then read through an information sheet, signed a consent form and were informed of their right to withdraw without penalty.

Participants were randomly assigned to one of the two Intervention groups: walking exercise group (*n* = 12), and the control group (*n* = 12). Randomization was achieved by assigning each individual a participant identification number between 1 and 24, each ID number was randomly assigned the digit one or two for respective groups in equal proportions (1: walking exercise group, 2: control group). The experiment was split into session 1 and session 2, in-between the sessions the participants either completed the moderate walking exercise or had 30 min to relax (control group). The order in which the SST’s were administered were counterbalanced (load or non-load).

##### Session 1

All participants completed the state and trait versions of the STAI to measure self-reported anxiety before the experimental and control condition to establish an accurate baseline. All participants then did the practice SST and completed the first and second measures of interpretation bias (SST) both cognitively loaded and non-loaded which they had 4 min to do each set of 20 sentences.

##### Intervention

The exercise group spent 30 min walking at a brisk pace around the University grounds, and then were invited back into the experiment lab. The control group spent the 30-min period in the experiment lab, the only stipulation was to refrain from internet use, social media, and not use their mobile phones.

##### Session 2

All participants then completed different versions of the SST (loaded and non-loaded) followed by the STAI. Once completed each participant was thanked for their participation and debriefed. Participants were provided with a copy of the debrief information, which outlined the aims of the study and how all participant data is anonymized and kept securely. They were provided contact details of the primary researcher in the instance they would like more information about the study or would like to withdraw from the study post-participation.

#### Data Analytic Approach

A mixed measures 2 × 2 × 2 mixed factorial ANOVA was used to analyze the data collected to investigate; Load/No Load × Walking Exercise/No Exercise × Session 1/Session 2. The within-subjects variable is whether the SST was cognitively loaded/non-loaded, and session 1/session 2, while the between-subjects factor is the moderate exercise/control group. A 2 × 2 ANOVA was used to measure the effect of moderate intensity exercise on state and trait anxiety. The within-subjects factor is session 1/session 2, while the between-subjects factor is the exercise/control group. Any significant effects found will be further investigated with Tukey HSD *post hoc* tests.

### Results

#### Preliminary Analysis

Before the full analysis was conducted, preliminary analysis of the data was conducted to investigate the assumptions of parametric tests; the data was assessed for outliers using box plot graphs, no outliers were identified. The histogram of standardized residuals indicated that the data contained approximately normally distributed errors, as did the normal P–P plot of standardized residuals, which showed points that were close to the line of fit. Levene’s test of homogeneity of variance was also used and found for all measures to be non-significant and therefore did not violate the assumption of homogeneity of variance. The scatterplot of standardized residuals showed that the data met the assumptions of homogeneity of variance and linearity. The Box’s test was insignificant for the analysis of experimental group, loading, and time on the SST’s, and was also insignificant for the STAI measures. The assumptions of parametric data were not violated, therefore parametric analyses could be performed.

#### Main Effects

There was a significant effect of pre and post measures of state anxiety measures *F*(1, 23) = 10.17, *p* < 0.005, η_p_^2^= 0.316, but no significant main effect of intervention group *F*(1, 23) = 2.87, *p* = 0.10 (**Table 1**). The interaction between session and intervention group was statistically significant for trait anxiety, *F*(1, 23) = 5.02, *p* < 0.05, η_p_^2^= 0.186 (**Table 1**). A Tukey HSD *post hoc* test confirmed that participants in the walking exercise condition significantly decreased in trait anxiety measures (*SE* = 0.72), *p* < 0.01. There was no significant effect of the intervention on interpretation bias scores (SST) *F*(1, 23) = 0.37, *p* = 0.54.

### Discussion

The findings from Study 1 suggests a significant decrease in self-reported trait anxiety measures when participants underwent a single session walking exercise protocol. However, the hypothesis is only partially supported, because there was no significant effect of walking exercise on state anxiety measures relative to the control condition. Furthermore, walking exercise had no significant effect on negative interpretation bias measures, both cognitively loaded and non-loaded, relative to the control group. The reduction in self-reported state anxiety supports the hypothesis, however, the effect was not relevant to group, therefore this rests on a reduction in state anxiety over time and one cannot assume this was due to the walking exercise intervention. There was a significant reduction in trait anxiety relative to the intervention group, from this we can suggest that the walking exercise had a positive effect on mood and in turn reduced individuals self-reported trait anxiety. This was in comparison to the control group which as hypothesized did not decrease in self-report trait anxiety measures, suggesting that a rest control condition does not improve mood state. This holds promise for the foundations of this research, which was the anxiolytic affect that physical activity has for anxiety and mood enhancement in general.

These findings suggest that as little as 30 min of low intensity exercise such as walking can have a positive effect on mental wellbeing, by reducing self-reported trait anxiety. However, it would seem that more moderate cardiovascular exercise is required to affect an individual’s cognitive interpretation biases.

## Study 2

### Introduction

Research has previously investigated the role of cognitive interpretation biases in stress reactivity (Mackintosh et al., 2013; Joormann et al., 2015). However, research to date has not investigated the effect of cognitive interpretation bias on perceived psychological stress as an independent factor, despite their being support for a link between clinical depression and perceived stress (Joormann and Quinn, 2014; Joormann et al., 2015). Indeed, our understanding of which psychological mechanisms mediate stress and how they affect mental health has been unclear (Creswell et al., 2005). Stress is a physiological disruption caused by tangible or subjective threats, which disrupt an individual’s physical or psychological state and may be caused by a single or combined physical, physiological, and psychosocial conditions (Sapolsky, 2004; Iwasaki, 2006). The stress response through the hypothalamic–pituitary–adrenal axis is thought to be a major physiological mechanism in which stress influences one’s health and cognition (Cohen et al., 2007; Norman et al., 2011). Previous research has also found that increased perceived psychological stress correlates with depression (Mazure, 1998; Hammen, 2005). Perceived levels of stress are suggested to be an important factor when studying the effects of exercise on cognitive biases (Salmon, 2001). Kajtna et al. (2011) investigated the effect of mood states on experienced levels of stress, they found no significant correlation between high experienced levels of stress and negative mood-states. This suggests that the physiological effect of stress has no effect on negative emotions and perhaps no effect on cognitive biases. This suggests support for the concept that mood enhancement of which elicited form exercise will not affect an individual’s cognitive bias, while there is also support to the contrary (Hallion and Ruscio, 2011). Furthermore, various studies have found support for the notion that those who suffer with clinical depression preferentially encode threatening interpretations (Mogg and Bradley, 2006; Mogg and Bradley, 2016). However, the evidence is not clear-cut and others have found no such evidence of negative interpretation bias in depressed individuals (Bisson and Sears, 2007).

Research into the effects of exercise training on cognitive interpretation biases in anxiety, depression and stress-related disorders is sparse and the insight it could lend to our understanding of the cognitive mechanisms could be valuable. The key aims of the present study is to establish at which intensity of exercise is required to achieve anxiety reduction and whether this is related to changes in an individual’s negative cognitive interpretation biases. Furthermore, it will investigate whether moderate exercise reduces self-reported symptoms of depression and stress. These research aims will be addressed with two experimental studies. The second study hypothesizes is that there will be significant decrease in negative interpretation bias while cognitively loaded, and a reduction in depression, stress, and anxiety measures in the moderate exercise condition relative to the control condition.

The key aims of the present study is to establish at which intensity of exercise is required to achieve anxiety reduction and whether this is related to changes in an individual’s negative cognitive interpretation biases. Furthermore, it will investigate whether moderate exercise reduces self-reported symptoms of anxiety, depression and stress. The hypothesizes for this second experimental study is that there will be significant decrease in negative interpretation bias while cognitively loaded, and a reduction in depression, stress, and anxiety measures in the moderate exercise condition relative to the control condition.

### Methodology

#### Participants

Forty-eight participants were recruited to participate in the study from a general population sample at a private gym in which they were members and randomly assigned to either an exercise or non-exercise (control) group (see **Table 2** for mean age, gender ratio, and mean HR rest). Randomization was achieved by assigning each individual a participant identification number between 1 and 48, each ID number was randomly assigned the digit one or two for respective groups in equal proportions (1: exercise group, 2: control group). Participants were recruited from and the experiment was conducted in, a private members gym in Colchester, United Kingdom. Eligibility requirements were that individuals must refrain from cardiovascular exercise 12 h prior to the experiment (regardless of the intensity) and they must be fluent in spoken and written English. Participants had to be over 18 years old and be regular gym users (twice a week or more) in order to be eligible. Participants were made told that the research had no affiliation with the gym, nor was there any obligation for them to participate. Ethical approval was granted from the University of Essex Ethics Committee.

**Table 2:** Study 2: Means and standard deviations for STAI, PSS, BDI-II, and SST measures for moderate exercise and control condition.

	Baseline: Session 1 Post condition: Session 2	*N*	Mean, range, and *SD* age	Gender ratio M:F	Moderate exercise condition Mean (*SD*)	Control condition Mean (*SD*)
STAI-S	Session 1	24	Exercise Group:	Exercise Group: 10:14	46.33 (12.49)	38.75 (6.74)
			Mean: 27.79			
			Range: 20–47			
			*SD*: 7.30			
	Session 2	24			31.04 (7.6)	39.58 (5.61)
STAI-T	Session 1	24			44.42 (7.87)	38.42 (7.87)
	Session 2	24			33.33 (10.70)	40.00 (6.20)
PSS	Session 1	24	Control Group: Mean: 25.66 Range: 19–42	Control Group: 9:15	31.75 (5.11)	25.21 (6.46)
			*SD*: 6.22			
	Session 2	24			22.29 (4.51)	22.54 (5.70)
BDI-II	Session 1	24			17.63 (7.44)	12.46 (5.70)
	Session 2	24			6.58 (3.85)	11.88 (5.60)
SST Cognitively loaded	Session 1	20	Exercise Group: *N* = 8	Exercise Group: 1:3	46.66 (16.63)	45.37 (16.97)
			Mean: 30.12			
			Range: 20–47			
			*SD*: 10.65			
	Session 2	20			42.77 (33.13)	52.92 (22.38)
SST Cognitively non-loaded	Session 1	20	Control Group: *N* = 12	Control Group: 5:7	79.27 (17.39)	52.18 (25.75)
			Mean: 26.91			
			Range: 19–38			
			*SD*: 5.88			
	Session 2	20			14.53 (6.47)	45.29 (20.01)


#### Materials

State and Trait Anxiety and Cognitive interpretation bias (SST) were measured as in Study 1.

##### Depressive symptoms

The revised Beck Depression Inventory (BDI-II; Beck et al., 1996) was used in this research because it measures attitudes, characteristics, and symptoms associated with depression. It is a 21 item inventory measured with a 4-point Likert scale. The BDI-11 score is calculated by totaling the answers from each questionnaire and scores of above 30 are indicative of severe depression.

##### Perceived psychological stress

The Perceived Stress Scale (PSS; Cohen et al., 1983) was used to measure how stressed the participant perceived themselves as being over the past 2 months. Responses on the 14-point scale are given on a rating of one (never) to four (always). The PSS contains questions such as “How often in the past 2 months have you dealt successfully with irritating life hassles?” and is used to measure an individual’s perception of how they are coping with stressors. Furthermore, this suggests that due to current circumstances an individual’s perception of their level of coping with stressors can change sporadically.

#### Design

This experiment used a mixed measures 2 × 2 × 2 factorial design; which will be used to analyze the data collected to investigate hypothesis one; Load/Non-Load × Exercise/ControlGroup × Session1/Session2. The within-subjects variable is the cognitively loaded or non-loaded SST, and session 1/session 2, while the between-subjects factor is the exercise/control group. The data analysis approach will use a 2 × 2 mixed factorial design; in which the within-subjects factor is Session1/Session2 (before and after experimental/control condition), and the between-subjects factor is exercise/control group. The dependent variables are the self-report measures of anxiety (STAI), stress (PSS), depression (BDI-II), and interpretation bias (SST).

#### Procedure

Once participants were recruited and deemed eligible for the study, they were invited to a study appointment with the primary researcher. The researcher summarized the process of the experiment and what would be required of them for participation. Participants then read through an information sheet, signed a consent form and were informed of their right to withdraw without penalty.

Participants were randomly assigned to the intervention group (*n* = 24) or the control group (*n* = 24) the experiment was split into session 1 and session 2, in-between the sessions the participants either completed the moderate intensity exercise protocol or had 40 min to relax.

##### Session 1

Participants completed the STAI, BDI-II, and PSS. They then had 4 min to complete each SST (the order of which were counter balanced between subjects). Although participants were for the majority randomly assigned to groups, a running tally was kept of gender and the state and trait anxiety scores (STAI) from session one, to allow for a homogenized sample in both the experimental and control group.

##### Intervention

Participants were assigned to either the experimental condition (a moderate exercise protocol) or the control condition, in which the participants had 40 min to relax until the second session of the experiment. The exercise protocol was compliant to current health and safety regulations as set out by the gym being used. The duration and intensity used was similar to previous research (e.g., Hansen et al., 2001; Russell, 2003), and based on findings that advocates that 85% of the cardiovascular aerobic heart rate reserve of each individual should be maintained for 20–30 min to achieve the desired benefits of aerobic activity (Barnes et al., 2010), which was relevant for the aims of this research. This research used a static cycle machine (York-Fitness-Model-110) in the gymnasium, as this reduces impact on joints and is safest for the participant, as they remain seated. The static-cycle machine was used to measure heart rate so that the desired aerobic heart rate that is necessary for the experiment can be monitored and adhered to respectively. This provided participants with continuous feedback of their heart rate reserve and when to increase/decrease their aerobic intensity. Heart rate monitors are recognized to be a reliable and valid measure of measuring exercise intensity and rate of exertion during aerobic exercise (Janz, 2002). Aerobic heart rate reserve was calculated using Eq. (2) (Uth et al., 2004). The exercise protocol consisted of a light warm up of low-intensity; which is 20% of heart rate reserve for 5 min, and then increased to 80% heart rate reserve for 30 min, then concluded with a ‘cool down’ which was low-intensity pedaling for 5 min using 20% heart rate reserve.

(2) $$\begin{array}{l} HR\;reserve=HR\;resting-HR\max imum\\ Z=80\%\;of\;reserve\\ Z+HR\;resting=Aerobic\;Heart\;Rate \end{array}$$

##### Session 2

Participants were invited back into the private consultation room to complete the third and fourth SSTs (counterbalanced between subjects) followed by the STAI, BDI-II, and PSS questionnaires. Once completed each participant was thanked for their participation and debriefed. Participants were provided with a copy of the debrief information, which outlined the aims of the study and how all participant data is anonymized and kept securely. They were provided contact details of the primary researcher in the instance they would like more information about the study or would like to withdraw from the study post-participation.

#### Data Analytic Approach

A mixed measures 2 × 2 × 2 mixed factorial ANOVA was used to analyze the data collected to investigate hypothesis one; Load/No Load × Exercise/No Exercise × Session1/Session2. The within-subjects variable is whether the SST was cognitively loaded/non-loaded, and session 1/session 2, while the between-subjects factor is the moderate exercise/control group. A 2 × 2 ANOVA was used to measure the effect of moderate intensity exercise on anxiety, depression, and perceived levels of psychological stress. The within-subjects factor is session 1/session 2, while the between-subjects factor is the exercise/control group. Any significant effects found will be further investigated with Tukey HSD *post hoc* tests.

### Results

#### Preliminary Analysis

Before the full analysis was conducted, preliminary analysis of the data was conducted to investigate the assumptions of parametric tests; the data was assessed for outliers using box plot graphs, there were outliers for STAI-State, STAI-Trait, and BDI-II. Due to a modest sample size it was deemed appropriate to leave the outliers in.

The histogram of standardized residuals indicated that the data for SST, STAI-State, STAI-Trait, BDI-II, and PSS contained approximately normally distributed errors, as did the normal P–P plot of standardized residuals, which showed points that were close to the line of fit. In respect that Kolmogorov–Smirinov test showed several dependent variables to not be normally distributed, therefore skew and kurtosis *z*-scores were calculated to investigate any positive or negative skew in the data set. The skew and kurtosis *z*-scores calculated showed no obvious positive or negative skew to the data. Levene’s test of homogeneity of variance was also used and found for all measures to be non-significant and therefore did not violate the assumption of homogeneity of variance.

The scatterplot of standardized residuals showed that the data met the assumptions of homogeneity of variance and linearity. The Box’s test was insignificant for the analysis of experimental group, loading, and time on the SST’s, and was also insignificant for the STAI, BDI-II, and PSS measures. The assumptions of parametric data were not violated, therefore parametric analyses could be performed.

#### Main Effects

Individuals who participated in the exercise group decreased in both state and trait anxiety measures, while the control group remained stable from session 1 to session 2 (**Table 2**). This was exemplified by a significant interaction between session and intervention group reducing state anxiety measures, *F*(1, 46) = 43.78, *p* < 0.005, η_p_^2^= 0.488, and reducing trait anxiety measures, *F*(1, 46) = 39.54, *p* < 0.005, η_p_^2^= 0.462 (**Table 2**). Tukey HSD *post hoc* tests revealed that participants in the exercise group significantly decreased in state anxiety measures (*SE* = 1.21), *p* < 0.001. Tukey HSD *post hoc* tests also confirmed that participants in the moderate exercise condition significantly decreased in trait anxiety measures also (*SE* = 1.0), *p* < 0.01.

Likewise, there was a significant decrease in self-report stress measures in the exercise condition relative to the control condition, and this was a significant interaction between session and intervention group, *F*(1, 46) = 11.24, *p* < 0.005, η_p_^2^= 0.196 (**Table 2**). Tukey HSD *post hoc* tests reveal a significant decrease in stress measures in the exercise condition (*SE* = 1.10), *p* < 0.05. Participants who underwent the moderate exercise protocol also decreased in self-report depression measures, the interaction between session and intervention group was significant *F*(1, 46) = 48.81, *p* < 0.005, η_p_^2^= 0.515 (**Table 2**). Tukey HSD *post hoc* tests support this finding of a significant decrease in depression measures in the exercise condition (*SE* = 0.74), *p* < 0.001.

There was a no significant reduction in interpretation bias scores in either the exercise or control condition, regardless of whether the SST was cognitively loaded or cognitively non-loaded *F*(1, 16) = 3.25, *p* = 0.09 (**Table 2**). Considering the modest sample size and that the main effect is not vastly distant from being statistically significant, the SST was then analyzed combining both cognitively loaded and non-loaded versions of the measure and there was a significant interaction between intervention group and session, *F*(1, 43) = 24.11, *p* < 0.005, η_p_^2^= 0.359. Tukey HSD *post hoc* tests suggest that for individuals who participated in moderate intensity exercise, there was significant decrease in negative interpretation bias from baseline to post exercise (*SE* = 3.80), *p* < 0.05.

### Discussion

The findings from Study 2 suggest that individuals who underwent the moderate exercise protocol reported significantly lower on both state and trait anxiety measures, relative to those in the control condition. Furthermore, participants in the moderate exercise condition also reported a significant decrease in depression and stress-related symptoms relative to those in the control condition. Participants who underwent the moderate exercise protocol also reduced in negative interpretation bias scores relative to controls, however, this was not specific to the cognitively loaded condition, which was specified in the hypothesis. When both cognitively loaded and non-loaded conditions were analyzed combined, it suggested that participants in the moderate exercise condition presented less negative cognitive interpretation biases from baseline measures, relative to participants in the control condition.

## General Discussion

The purpose of Study 1 was to further investigate the functions of exercise and mood enhancement, with a focus of anxiety. The aim was to further our understanding of the relationship between exercise regimes and self-report anxiety. The interest primarily was to investigate whether one single session of exercise is sufficient to produce the anxiolytic effects that is well founded. Furthermore, to investigate whether less intense exercise such as walking can produce the same mood enhancing effects as a more intense cardiovascular exercise session. The present research also investigated whether a cognitive load manipulation could have an effect on individual’s interpretation biases, and whether this produced a more positive or negative representation of an individual’s interpretation biases. Study 2 aimed to investigate, whether moderate intensity physical exercise was necessary to effect cognitive interpretation biases and whether this could in turn reduce reported symptoms of stress and depression, as well as anxiety. Study 2 also employed a cognitive load manipulation to measure whether individual’s cognitive biases could in fact be affected by a single bout of moderate physical exercise.

Needless to say, there is an abundance of research focusing on the longer term effects of physical exercise for reducing self-report anxiety, however, the results of this present study do highlight some mood enhancing effects after a single session of physical exercise, these effects seem to be more so with moderate intensity exercise compared to light intensity physical exercise. The investigation of cognitive interpretation biases was made using the SST, which is administered both cognitively loaded and non-loaded. It takes concentration to remember the six-digit number, which decreases the amount of concentration an individual has while performing the task, therefore their attention is distracted from these threat-engaging thoughts which provoke negative interpretation biases. The underlying logic of this cognitive loading manipulation being that individuals would find it difficult to suppress these negative thoughts and interpretations during the cognitive load manipulation, and therefore the cognitive load measure would be a more accurate representation of an individual cognitive interpretation bias in comparison to the non-cognitively loaded measure. However, the cognitive load manipulation did not significantly affect whether the participants presented more positive or negative interpretation biases in either Study 1 or Study 2.

Both studies together lend valuable insights from multiple perspectives. They offer further understanding into the relationship between physical exercise and anxiety reduction on the ground that regardless of the mode of exercise employed (physical activity, physical aerobic exercise), there is arguably growing support for the connection between physical exercise and mood enhancement. However, the nature of the methodological design of these two studies (within-subjects) provided sufficient statistical rigor for the purpose of these two studies. These studies were designed as analog studies, so it becomes important for the betterment of our understanding to replicate these studies with a clinical or subclinical sample, as currently this cannot be generalized to a wider population. There could be a greater improvement in these individuals as the scope for reduction in anxiety and depression could be increased. Whether future research employing similar methodology to that of these two studies are able to replicate these findings or larger sample sizes differ in responses across conditions could become more pronounced, remains to be resolved.

## Conclusion

The present studies have built upon the current literature and have lent support to claims that moderate intensity walking and aerobic exercise ameliorate the symptoms of anxiety, depression, and psychological stress. In Study 2, there were signs that SST (interpretation) could also be changed. However, the present research investigated a non-clinical sample that have no previously diagnosed mental health conditions, therefore to what extent this research can be applied holds promise, but indeed is limited. Given the exciting potential for the use of exercise and cognitive bias modification in the clinical interventions, more research into this emerging field is clearly warranted.

## Author Contributions

SC responsible for data collection, analysis, production of original research article. NC responsible for production and editing of original research article. MR responsible for data collection. BM responsible for research design, methodology, and editing of original research article.

## Conflict of Interest Statement

The authors declare that the research was conducted in the absence of any commercial or financial relationships that could be construed as a potential conflict of interest.
